# Effectiveness of cognitive rehearsal programs for the prevention of workplace bullying among hospital nurses: a systematic review and meta-analysis

**DOI:** 10.1186/s12889-024-18969-x

**Published:** 2024-06-11

**Authors:** Yulliana Jeong, Hye Sun Jung, Eun Mi Baek

**Affiliations:** Department of Preventive Medicine, College of Medicine, Catholic University of Korea, 222 Banpo-daero, Seocho-Gu, Seoul, 06591 South Korea

**Keywords:** Systematic review, Nurse, Bullying, Cognitive rehearsal program, Meta-analysis

## Abstract

**Background:**

To solve the problem of workplace bullying among nurses, it is necessary to review the effects of interventions and generalize the findings. We conducted a systematic literature review and meta-analysis to evaluate the effects of cognitive rehearsal programs on workplace bullying among hospital nurses.

**Methods:**

Data were collected from March 30 to April 11, 2021, and 11,048 journal articles published in South Korea and internationally were examined across eight databases. Nine articles were selected for inclusion in the systematic literature review; five of the nine studies were included in the meta-analysis. For randomized controlled trials, the risk of bias was evaluated, and for non-randomized controlled trials, the study quality was evaluated using the Risk of Bias for Non-randomized Studies version 2.0. Egger’s regression test was performed to determine publication bias.

**Results:**

Of the nine articles selected for this study, two were randomized controlled trials and seven were non-randomized controlled trials. The I^2^ value was 18.9%, indicating non-significant heterogeneity. The overall effect size of the cognitive rehearsal programs was -0.40 (95% confidence interval: -0.604 to -0.196; Z = -3.85; *p* = .0001) in a random-effects model, indicating a large effect size with statistical significance.

**Conclusions:**

Therefore, cognitive rehearsal programs that address workplace bullying among hospital nurses are effective. Health policymakers must implement cognitive rehearsal programs in a policy manner to address the problems of bullying in the workplace.

**Supplementary Information:**

The online version contains supplementary material available at 10.1186/s12889-024-18969-x.

## Background

In 2018, a nurse in South Korea committed suicide, sparking a national conversation among hospital nurses regarding workplace bullying as a social issue. In response, the government revised article 76.2 of the Labor Standards Act and enacted the Workplace Anti-Bullying Law [1]. Despite these governmental measures, various forms of workplace bullying have become institutionalized and persist among nurses [2, 3]. Accordingly, workplace bullying remains a serious issue in clinical nursing [4].

Regarding definition, workplace bullying refers to repeated behaviors that make coworkers feel aggravated, uncomfortable, or socially isolated in the workplace over a sustained period of time [5]. In the context of hospitals in South Korea, this type of bullying is referred to either as “bullying” [6], which comes from the English word for aggressions targeted toward individuals, or “mobbing” [7, 8], meaning aggressions targeted toward groups. Various other terms have been used as synonyms, including “workplace bullying,” “workplace harassment,” and “moral harassment” [9].

Prior research has reported that workplace bullying is more common in hierarchical organizations with power imbalances [10]. Nursing organizations can be hierarchical because senior nurses typically educate apprentices in a high-stress environment in which the lives of patients are at stake [11]. In such environments, bullying has been normalized as part of the educational process, and scholars have shown that bullying is reported more often in the nursing field than in other occupational fields [5, 12, 13]. For example, compared to other hospital-based occupations, such as radiologists, physical therapists, and doctors, nurses reported a higher prevalence of bullying [14–16]. Furthermore, the prevalence of workplace bullying ranged from 5–36% among nurses in Scandinavia, the United Kingdom, and the United States of America; from 50–57% in Australia; and the prevalence was 86.5% in Turkey [17]. Among Japanese nurses, this prevalence was 18% [18]. In Sweden, the prevalence range was from 4.8 to 31.4% [19]. In a study conducted in the United Kingdom, over 80% of the nurses experienced bullying [20]. These pieces of evidence indicate that workplace bullying among nurses is common across cultures.

Nurses who face workplace bullying can experience physical and psychological symptoms, including depression [21, 22], anxiety, insomnia [23], and post-traumatic stress syndrome [24]. These experiences, in turn, result in increased work-related stress, burnout, and turnover intention, as well as decreased work satisfaction [25]. These outcomes can negatively affect organizational productivity, social costs, medical errors, and patient safety [26–28]. Therefore, workplace bullying can have an impact not only at the individual but also at the organizational and social levels. This makes it important to establish preventive strategies for tackling workplace bullying before it can begin to take shape.

Academicians have conducted various studies on workplace bullying among hospital nurses, but a clear and consistent solution to the phenomenon has yet to be identified [29]. A prior study on research trends identified that the most frequently studied topics regarding workplace bullying in South Korea were, in descending order of frequency, “intention to quit,” “organizational culture,” “tools,” and “effects” [30]. This shows that studies on workplace bullying tend to focus on the outcomes of the phenomenon. Studies on interventions to reduce bullying are limited. It also implies that the number of intervention studies is currently relatively limited, and the need for more research efforts to review the effects of existing interventions and enable a greater generalization of their application.

In the current literature, interventions to prevent workplace bullying include cognitive rehearsal programs [31–35], transition programs for new nurses, mentoring programs, self-assertion training, and educational programs [36]. Specifically, Stagg and Sheridan [29] suggested cognitive rehearsal as a method for identifying and responding to workplace bullying. This method was first developed by Griffin [31] as an intervention to alleviate workplace bullying among nurses. It is a form of cognitive-behavioral therapy in which interactions or coping processes are practiced by recreating specific situations [37].

Cognitive rehearsal is an effective tool for intervention programs because it has been shown to enable nurses to increase their knowledge and awareness of workplace bullying and respond to related conflicts using previously rehearsed methods. One study that reviewed the effect of cognitive rehearsal on workplace bullying among nurses reported heterogeneity in the results of the included studies. While some studies reported that cognitive rehearsal interventions have no effect on the incidence of workplace bullying among nurses [32, 36], others showed that such interventions increase awareness of workplace bullying and effectively reduce it [38]. As previous studies provide heterogeneous evidence for the effect of cognitive rehearsal on workplace bullying among nurses, each study should be systematically and comprehensively reviewed.

Accordingly, this study aimed to analyze the characteristics and contents of cognitive rehearsal programs for the prevention of workplace bullying among hospital nurses and examine their effectiveness through a systematic literature review and meta-analysis. The specific aims of this study were as follows:

1. To understand the general characteristics of the body of literature on cognitive rehearsal programs for the prevention of workplace bullying among hospital nurses.
2. To assess the methodological quality of the studies included in the meta-analysis and understand the characteristics of the cognitive rehearsal programs applied in these studies.
3. To analyze the total effect size of the cognitive rehearsal programs used in the meta-analyzed studies to understand their effectiveness in preventing workplace bullying among hospital nurses.

By assessing the effectiveness of these programs on workplace bullying among hospital nurses, this systematic literature review and meta-analysis showed that the cognitive rehearsal programs used in the five meta-analyzed studies were generally effective.

## Materials and methods

### Study design

This study is a systematic review and meta-analysis aimed to examine the effectiveness of cognitive rehearsal programs in preventing workplace bullying among hospital nurses. The manuscript is reported following the Preferred Reporting Items for Systematic Reviews and Meta-Analysis guidelines [39].

### Inclusion and exclusion criteria

The main research topic in the literature selection process was the effectiveness of cognitive rehearsal programs in preventing workplace bullying among hospital nurses. The inclusion criteria of studies were based on the Populations, Interventions, Comparisons, Outcomes framework devised by the Cochrane Collaboration group for systematic reviews [40]. This framework was selected for use in this systematic review through discussions between the two of the authors. Search terms were constructed by focusing on populations (P) and interventions (I), whereas comparisons (C) and outcomes (O) were not specified nor limited. The population of this study was “hospital nurses” and the interventions were “cognitive rehearsal programs.”

#### Inclusion criteria

1. Studies that included hospital nurses;
2. mentioned a cognitive rehearsal program;
3. in which all mentioned departments were related to hospital-based clinical practice;
4. on interventions to address workplace bullying among nurses.
5. Study design types include intervention studies with original articles, systematic reviews and meta-analyses
6. Studies published since 2000.

#### Exclusion criteria

1. News articles or articles published in a letter format;
2. studies for which the full text was unavailable;
3. studies published in a language other than English or Korean;
4. positional statements of professional associations;
5. studies on bullying by patients, caregivers, doctors, and other employees;
6. gray literature (i.e., conference presentations, abstracts only, dissertations, and opinions).

### Search methods

This study followed the identification, screening, and inclusion processes mentioned in the flow diagram for systematic reviews of the Preferred Reporting Items for Systematic Reviews and Meta-Analysis guidelines [39] to select the studies included in the final analysis. The literature search and data collection procedures were conducted under the guidance of an information retrieval expert with 20 years of experience.

The literature search was conducted using electronic databases and other methods. Based on the Core, Standard, Ideal (COSI) model [41], the international databases of EMBASE (Elsevier), Cochrane Library, CINAHL, and PubMed were searched. The search expressions are detailed in Supplementary Material 1. For Korean articles, the Korean databases of Research Information Sharing Service, Korean Studies Information Service System, and Korean Medical Database–were searched. Medical Subject Headings (MeSH)-controlled vocabulary was used for PubMed and the Cochrane Library, and Emtree-controlled vocabulary was used for EMBASE. After controlling for vocabulary, natural language search terms were added. Boolean operators (AND, OR, and NOT) were used between search terms to formulate the search strings.

For the Korean studies, “간호사 괴롭힘” (“nurse bullying”), “간호사 무례함” (“nurse rudeness”), “간호사 태움” (“nurse burnout”), and “간호사 폭력” (“nurse violence”) were used in the search string. The search string for the international studies included “nurses,” “bullying,” “mobbing,” “rudeness,” “incivility,” “workplace incivility,” “lateral violence,” “vertical violence,” and “horizontal violence.”

### Literature selection

A total of 10,927 studies were identified from eight databases. Using the literature management software EndNote X20, duplicates were removed, and grey literature such as conference abstracts identified within EndNote were also excluded. This resulted in the removal of 3,927 records prior to screening. The remaining 6,910 studies had their titles and abstracts reviewed for relevance. Two researchers independently screened these documents, and any articles where suitability could not be determined by the abstract alone were subjected to full-text review. Out of these, 6,819 articles were excluded during this initial review phase because they either did not directly pertain to hospital nurses, were not original research (e.g., conference presentations), or did not align with the study’s thematic focus. Consequently, a total of 6,891 studies were excluded due to irrelevance, leaving 19 studies. These 19 studies underwent a full-text review and further exclusion criteria application, resulting in nine studies being selected for the systematic review. Of these, seven were suitable for qualitative assessment, and five contained quantitative data appropriate for inclusion in the meta-analysis. 7 of the 9 studies were qualitatively assessable and 5 studies included quantitative data and had comparable data, allowing for meta-analysis.

**Fig. 1:**
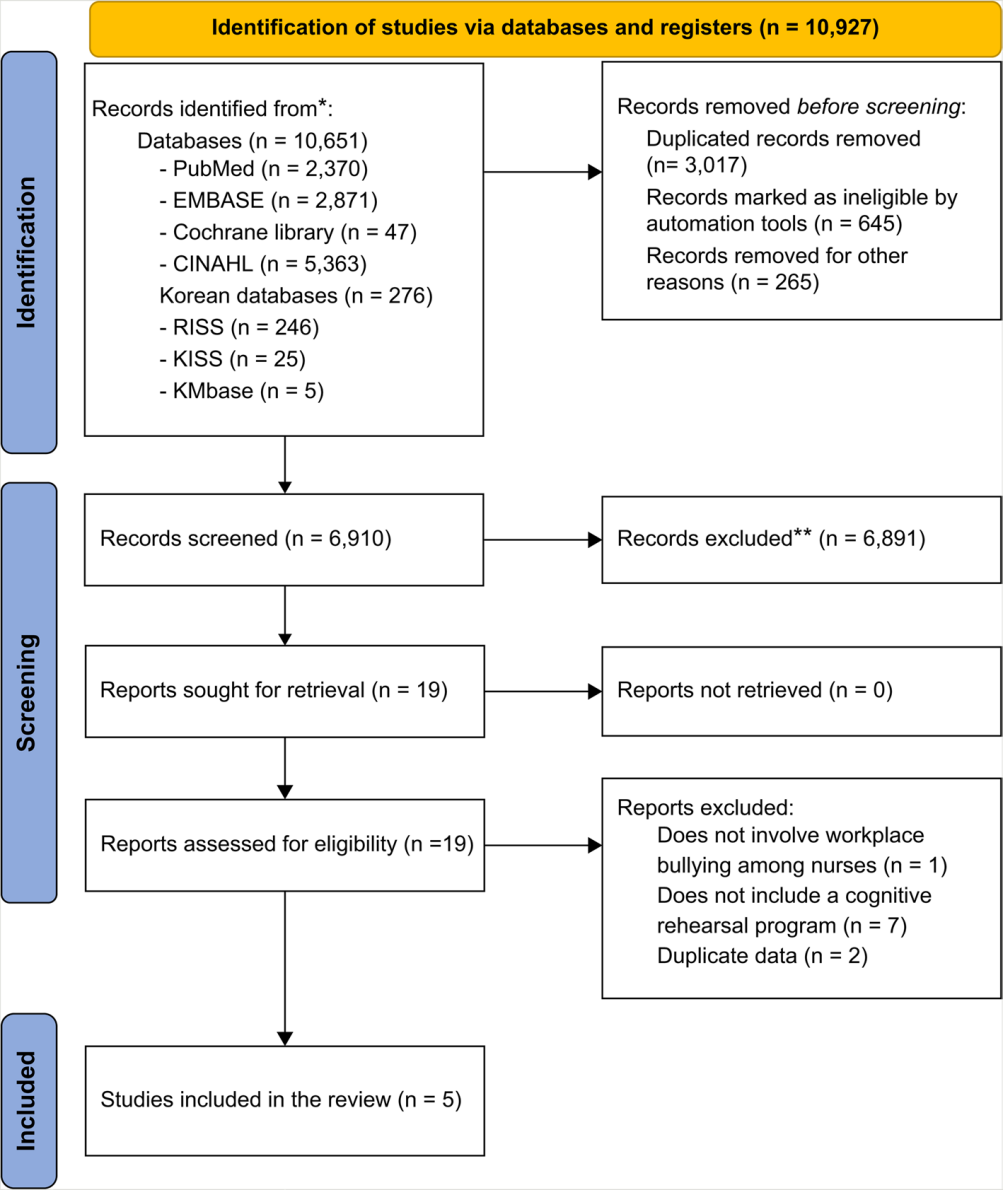
PRISMA flow diagram for literature search

Two researchers, Y.J. and E.M.B, who were also professors, developed the pre-determined inclusion criteria used in study selection, and conducted the selection process. One of the professors had over seven years of expertise in job stress and workplace bullying, and the other had over 10 years of experience in clinical hospitals and over three years of research experience In the first round of study selection, the titles and abstracts were reviewed to determine whether the studies met the inclusion criteria. When it was difficult to determine whether a study met the inclusion criteria based on the title and abstract, the study was moved on to the second round of study selection, in which the full text of the studies was reviewed. The final decision on study inclusion was made in the second round. The search results were collated and organized using a reference management software (EndNote, version 20; Fig. 1).

### Quality evaluation of the studies included in the systematic review

Randomized controlled trials (RCTs) were critically reviewed using the risk of bias (RoB) tool developed by the Cochrane Bias Method Group, and non-RCTs were evaluated using RoBANS 2.0 developed by the Korea National Evidence-based Healthcare Collaborating Agency [42]. The results of the quality evaluation were analyzed using RevMan 5.0 (Cochrane Community).

Quality assessment of seven of the nine studies included in the systematic review was conducted independently by two researchers (Y.J. and E.M.B). While nine studies were selected for the systematic review, only seven underwent quality assessment due to the specific design and focus of these studies, which made them suitable for comprehensive quality evaluation. The remaining two studies, though valuable for inclusion in the review for their relevant findings, did not meet the criteria set for detailed quality assessment based on their study design. If the researchers’ assessments diverged, a third researcher, who was a nursing professor, would intervene to resolve the divergence. However, there were no disagreements between the evaluations of the two independent reviewers.

### Data analysis

1. Regarding the general characteristics of studies on the effectiveness of cognitive rehearsal programs, they included country of origin, study design, sample size, cognitive rehearsal program components, intervention duration, intervention frequency, follow-up period, and main outcome variables.
2. The combined effect size of the cognitive rehearsal programs reported in the studies that were included in the meta-analysis was analyzed in R software after coding the data using Microsoft Excel. The details of the analysis are as follows:
  - a random-effects model was used for the meta-analysis, which assumed heterogeneity in the population, such as in study methods, sample, and intervention methods;
  - the combined effect size was interpreted using the standards devised by Cohen [43] for interpreting effect sizes. The statistical significance of each effect size was determined at a 95% confidence interval (CIs);
  - effect size heterogeneity was examined using *I*^*2*^ value, which refers to the proportion against the total distribution. Statistical tests for heterogeneity include the chi-square test (*Q* statistics) and Higgin's *I*^*2*^ statistic. *I*^*2*^ is a type of noise ratio, which is the proportion of the total variation in an observed effect that is due to heterogeneity [32]. *I*^*2*^ was chosen because, unlike *Q* statistics, it is insensitive to both scale and number of studies. For this study, we chose *I*^*2*^ because it is insensitive to the number of studies.

The difference between the effect sizes of the studies used in a meta-analysis is called heterogeneity. The degree of heterogeneity can be visually determined using a forest plot in Fig. 4 of Funnel plot of publication bias. These statistics include I^2^, the ratio of the actual between-study variance to the total variance. Heterogeneity is interpreted as small if it is 25% or less and medium between 25 and 75%. It is considered very large if it is 75% or more.

3) Funnel plots and Egger’s regression analysis were used to confirm publication bias.

## Results

### Characteristics of the studies included in the systematic review

This study analyzed the general characteristics of nine studies that reported on the application of a cognitive rehearsal program to mitigate workplace bullying among hospital nurses. All nine studies were published between 2004 and 2019 in academic journals. Two studies (22.2%) used a quasi-experimental pretest–posttest design, two (22.2%) were non-RCTs, two (22.2%) used a quasi-experimental post-test design, one (11.1%) was an RCT, one (11.1%) was a cluster quasi-randomized trial, and one (11.1%) was a mixed-method pilot study. The number of participants in the experimental and comparison groups ranged from 10–76. Seven of the nine studies (77.8%) were published in the United States of America and two (22.2%) were from South Korea. None of the comparison groups received any intervention, while the experimental groups attended cognitive rehearsal programs.

The duration of the cognitive rehearsal programs ranged from one to 20 sessions. The programs had the following formats: a 20-h program conducted across 10 sessions that included scenarios about bullying situations, standard communication, and role-play (1 study); 3-h programs (3 studies); a program that included a 30-min education session about horizontal violence and a 90-min participatory role-play intervention (1 study); an introduction to nonviolent conversation as a standard communication method that incorporated webtoons about workplace bullying situations (6 studies); a cognitive rehearsal intervention using a smartphone application with question-and-answer boards (1 study); a program that took place across five 2-h sessions over three weeks that included education about rudeness, cognitive rehearsal methods to respond to the top-10 types of rudeness, and a role-play intervention (1 study); a 1.5-h cognitive rehearsal education session with a focus on changing awareness (1 study); a 1-h education session on cognitive rehearsal followed by role-play (1 study). The number of sessions across studies was 1 (5 studies), 5 (1 study), and 10 sessions (1 study). In addition, one study described that there were multiple sessions and another did not specify the number of sessions.

The tools used in the studies included the Negative Acts Questionnaire-Revised (NAQ-R; 3 studies), Nurse Incivility Sale (NIS; 2 studies), tools based on the study by Griffin [31] (2 studies), the National Database of Nursing Quality Indicators (1 study), the Workplace Bullying Inventory (1 study), and others. Four studies used 2 or more tools (Table 1). Of the nine selected papers, two were systematic review papers and were not included in the meta-analysis. Systematic reviews, which analyze literature without quantifying data, were not included in this study because they did not meet the requirements for being included in a meta-analysis. The list of these two papers is presented in Supplementary Material 2.

**Table 1:** Description of the studies included in the systematic review (*N* = 9)

**Author (year) ref. no**	**Country**	**Study design**	**Participants**	**Intervention**		**Follow-up period**	**Main outcome variable (analysis tool)**	M*
				**CRP Components**	**Length/duration**			
Razzi and Bianchi (2019) [44]	USA	ㆍNon-RCT	ㆍ24 nurses	ㆍScripted phrases provided ㆍPreparing nurses to use common language to react to uncivil comments or behavior	1 h across multiple sessions	1 months	ㆍAwareness of the problem of incivility (NIS)	Y**
O’Connell et al. (2019) [45]	USA	ㆍNon-RCT	ㆍ76 pre-intervention ㆍ39 post-intervention	ㆍEducation on lateral violence followed by cognitive rehearsal training (modeled after the study by Griffin, 2004 [31])	2 h	3 months	ㆍPersonal bullying ㆍWork-related bullying ㆍPhysical intimidation (NAQ-R)	Y**
Kang and Jeong (2019) [34]	South Korea	ㆍQuasi-randomized cluster	ㆍ36 nurses in intervention group ㆍ36 nurses in comparison group	ㆍSmartphone application (non-violent conversation/6 webtoons depicting workplace bullying situations)	8 weeks	4/8 weeks	ㆍIndividual bullying ㆍWork-related bullying ㆍIntimidation-related bullying ㆍTurnover intention (NAQ-R)	Y**
Kile et al. (2019) [46]	USA	ㆍMixed-method pilot study	ㆍ17 nurses	ㆍReplicated the CRP IN Griffin’s [31] study ㆍ3 week (duration)	2 h in 5 sessions	6 weeks	ㆍRecognition of incivility ㆍAbility to confront incivility ㆍPerceived instances of incivility (NDNQI, NIS)	Y**
Kang et al. (2017) [32]	South Korea	ㆍRCT	ㆍ20 nurses in intervention group ㆍ20 nurses in comparison group	ㆍCRP stage (Role-playing/creating communication standards; Re-role-playing/feedback and evaluation)	20 h in 10 sessions	4 weeks	ㆍInterpersonal relationships ㆍTurnover intention ㆍWorkplace bullying ㆍSymptom experience (Relationship Change Scale, NAQ-R)	Y**
Dahlby et al. (2014) [36]	USA	ㆍQuasi-experimental pre-post test design	ㆍ29 nurses before intervention ㆍ25 nurses post-intervention	ㆍEducational intervention through cognitive rehearsal	1.5 h	12 weeks	ㆍCapacity to identify causes of bullying ㆍBullying prevalence (The lateral and vertical in nursing survey)	N***
Stagg et al. (2013) [38]	USA	ㆍQuasi-experimental posttest design	ㆍ10 nurses	ㆍTheoretical bullying ㆍBullying behaviors ㆍBullying consequences ㆍResponses to bullying ㆍCognitive rehearsal response	2 h	24 weeks	ㆍBullying ㆍAwareness of bullying ㆍResponse rate to bullying (created by Griffin, 2004 [31])	N***
Stagg et al. (2011) [47]	USA	ㆍQuasi-experimental pretest–posttest design	ㆍ15 nurses	ㆍTheoretical bullying ㆍBullying behaviors ㆍBullying consequences ㆍResponses to bullying ㆍCognitive rehearsal technique	2 h	Not reported	ㆍKnowledge of bullying management ㆍAwareness of bullying behaviors (WBI; Griffin, 2004 [31])	N***
Griffin (2004) [31]	USA	ㆍQuasi-experimental posttest design	ㆍ26 new nurses	ㆍTheoretical bullying ㆍBullying consequences ㆍResponses to bullying ㆍCognitive rehearsal technique ㆍThe 10 most frequent forms of lateral violence	2 h	More than 24 weeks	ㆍPrevalence of bullying ㆍHigh response rate to lateral violence (created in this study)	N***

*CRP* Cognitive rehearsal program, *NAQ-R* Negative Acts Questionnaire-Revised, *NDNQI* National Database of Nursing Quality Indicators, *NIS* Nurse Incivility Scale, *RCT* randomized controlled trial, *WBI* Workplace Bullying Inventory

M*: Applicable to meta-analysis, Y**: Yes, N***: No

### Quality appraisal of the studies included in the systematic review

We assessed the quality of seven of the nine included studies. Two RCTs were evaluated using the Cochrane RoB tool, and both (100%) showed uncertainty regarding randomization; a low risk of missing values; a low risk regarding the selection of the reported study results; and an uncertain risk regarding the measurement of intervention results. Deviation from the intended intervention was low in one study (50%) and high in another (50%). In the other RoB categories, one study (50%) had an uncertain risk and the other (50%) had a high risk.

**Fig. 2:**
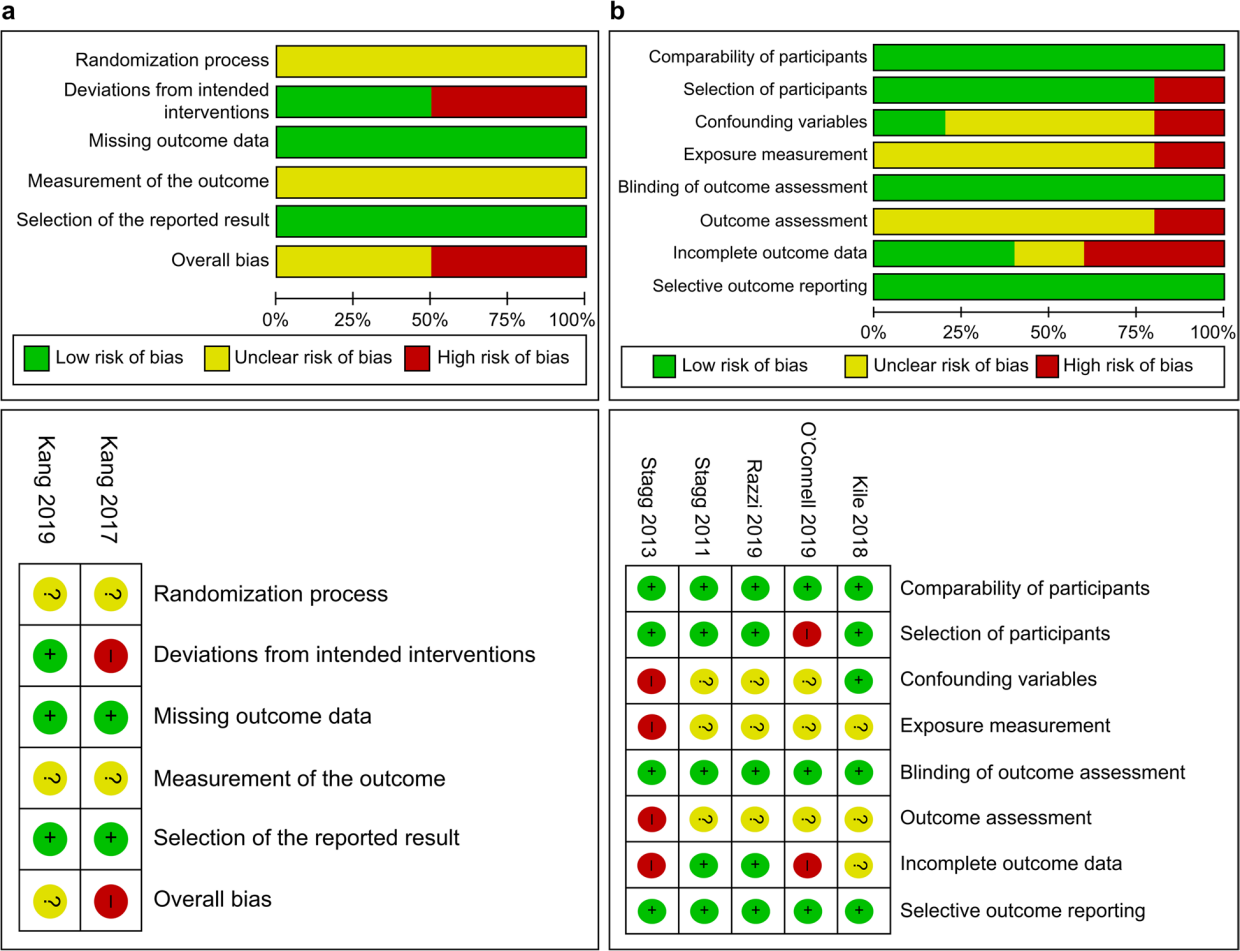
**A** Risk of bias graph (RoB 2.0). **B** Risk of bias graph (RoBANS 2.0)

The quality of the five non-RCT studies was evaluated using RoBANS 2.0. All five studies (100%) had a low RoB of participant comparability. Four studies (80%) had low bias and one study (20%) had high bias regarding the selection of a comparison group. Three studies (60%) had an uncertain, one (20%) had a high, and one (20%) had a low RoB regarding confounding variables. Four studies (80%) had an uncertain and one (20%) had a high RoB regarding exposure measurement. All five studies showed a low RoB for the blinding of the outcome assessment. Regarding the evaluation of the results, four studies (80%) had an uncertain RoB and one (20%) had a high RoB. Two studies (40%) had a low, two studies (40%) had a high, and one study (20%) had an uncertain RoB related to incomplete outcome data. All five studies (100%) had a low RoB concerning the selective outcome reporting criteria (Fig. 2).

### Meta-analysis of the effectiveness of the cognitive rehearsal programs

In the study included in the meta-analysis [32], The participants in the study were divided into two groups: the experimental group and the wait-list group. The allocation of participants to these groups was done using a random allocation table for two groups from Research Randomizer. In the study included in the meta-analysis [13], three groups were specified because the participants were all 72 hospital nurses working at a university hospital in South Korea. The three groups were intervention group, control group 1, and control group 2. The intervention group received a cognitive rehearsal intervention via a smartphone application that included common bullying situations and appropriate non-violent communication scenarios. Control group 1 received no intervention and control group 2 received a general health education program unrelated to workplace bullying.

In this study, we assessed the effects of cognitive rehearsal programs by examining their impact on various factors related to bullying, as previously identified in the literature. Specifically, the meta-analysis included multiple indicators from two papers [32, 34], and these were analyzed separately to ensure a detailed and nuanced understanding of the data. Kang [32] distinguished and measured two scales: workplace bullying and symptom experience. Our analysis separately reviewed the effects associated with these two variables. Furthermore, Kang [34] reported the primary effects of cognitive rehearsal programs by categorizing them into person, work, and intimidation categories. Despite being from the same sample, all three indicators were included in our analysis because they represent different dimensions of the CRP's impact that align with the objectives of our study, thus warranting their individual consideration.

The effect sizes of the cognitive rehearsal programs were calculated by analyzing the correlation coefficients using the number of participants in the experimental and control groups, means, and standard deviations. The results of the average effect size analysis are presented in Table 2.

**Table 2:** Analysis of the effect size of all variables

**Model**	**ESr**	**95% CI**	***z***	***p***	***I***^***2***^
Fixed	-0.406	-0.588; -0.225	-4.39	< .0001***	18.9
Random	-0.400	-0.604; -0.196	-3.85	.0001	

*CI* confidence interval

^***^p < .001, *Q* value = 8.63, *p* = .280

**Fig. 3:**
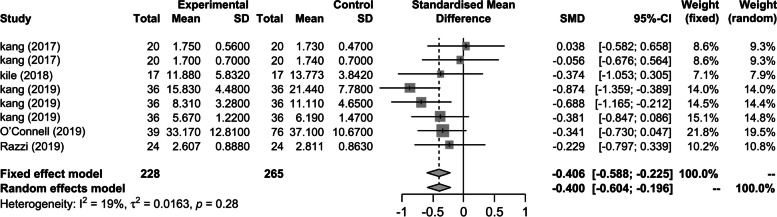
Forest plot of effect size; CI, confidence interval; SD, standard deviation

Results showed that the overall effect size of the fixed-effects model was -0.406 (95% CI: -0.588, -0.225; Z = -4.39; *p* < 0.0001), and that of the random-effects model was -0.40 (95% CI: -0.604, -0.196; Z = -3.85; *p* = 0.0001). In both models, the effect sizes were interpreted as large. The CIs from both models did not include zero, suggesting that the results were statistically significant. The I^2^ value for heterogeneity was 18.9%, indicating a low degree of heterogeneity (Fig. 3).

### Sensitivity analysis on CRP duration

To understand the influence of cognitive rehearsal program duration on the observed effect sizes across studies, a sensitivity analysis was conducted using regression modeling. This analysis aimed to determine whether longer durations of CRP are associated with greater effectiveness as measured by effect sizes (Hedges' g). A linear regression model was employed where CRP duration was the independent variable and Hedges' g was the dependent outcome. This analysis included data from the five studies with varying CRP durations ranging from 1 to 20 h. The model indicates a statistically significant positive relationship between the duration of CRP and the effect sizes. The positive slope (*β*_*1*_ = 0.0254) suggests that for each additional hour of CRP, there is an expected increase of 0.0254 in the Hedges' g value, holding other factors constant. This analysis underscores the importance of CRP duration in enhancing the effectiveness of interventions aimed at reducing workplace bullying among hospital nurses. Longer durations of CRP were associated with larger effect sizes, suggesting that more extended exposure to CRP may be beneficial in achieving more substantial improvements. Table 3 describes the results of sensitivity analysis.

**Table 3:** Sensitivity analysis

**Metrics**	**Intercept (*β0*)**	**Slope (*β1*)**	***P*-value**	***R***^***2***^	**Correlation coefficient**	**95% CI**
Value	-0.5253	0.0254	0.05	0.501	0.7077	0.00007, 0.0507

### Publication bias

**Fig. 4:**
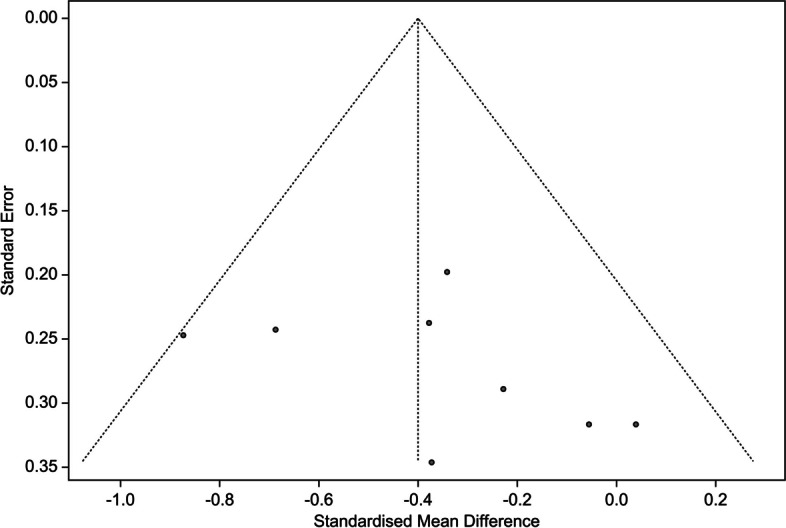
Funnel plot of publication bias

Funnel plot and Egger’s regression analysis were used to analyze the publication bias of the meta-analyzed studies. When visually analyzed using the funnel plot, we did not observe any obvious asymmetry in the distribution of effect sizes from the included studies (Fig. 4). There was no asymmetry detected, but it was deemed statistically insignificant by Egger regression analysis. The results indicated no publication bias (t = 1.1, *p* = 0.313).

## Discussion

This study aimed to analyze the effectiveness of cognitive rehearsal programs on workplace bullying among hospital nurses through a systematic review and meta-analysis of articles published in South Korea and internationally. Nine studies were included in the systematic review, and a meta-analysis was conducted with five (of the nine studies) studies that included quantitative data analysis results.

Among the nine studies included, seven were international, and two were from South Korea. This research initially reviewed existing literature related to bullying among nurses. According to [48], studies examining variables associated with workplace bullying among nurses included 23 studies, and according to [12], another research focusing on the prevalence of workplace bullying encompassed 14 studies. Based on the review results, it can be inferred that there is a scarcity of studies on cognitive rehearsal intervention programs aimed at addressing workplace bullying among hospital nurses.

This corroborates the argument in a study that examined South Korean research trends in workplace bullying among nurses that the number of intervention studies on the topic is insufficient [30]. Overall, the findings of the works we reviewed agree with the observations of previous studies that investigated the general bullying context [49] and bullying contexts in nursing [33, 50]. Recently, the Convention on the Prohibition of Workplace Violence and B ullying was adopted at the 108th Session of the International Labour Organization conference [51]. Furthermore, many countries, including South Korea, the Netherlands, Sweden, France, Belgium, Finland, Canada, Australia, Japan, and the United Kingdom have laws prohibiting workplace bullying [52]. Despite the existence of laws in many countries, the number of workplace bullying cases is increasing every year. These governmental measures reflect the increased interest in the effective mitigation of workplace bullying through systematic interventions. Still, in a hospital environment characterized by strong hierarchy, power imbalances, and high stress, nurses tend to assume relatively weak positions. Accordingly, researchers have described that the characteristics of nursing organizations [53] may explain why workplace bullying is more common among nurses than among other healthcare personnel [12]. These characteristics also support the point that laws designed to manage workplace bullying after its occurrence are insufficient to tackle workplace bullying effectively, and that preventative interventions are needed to eradicate this phenomenon.

The most frequently used tool in the included studies was the NAQ-R developed by Einarsen et al. [5], which was used in three studies. Two of these studies used a version of the NAQ-R translated and adapted to Korean by Nam et al. [54]. The NAQ-R is a shortened, English-language version of the NAQ developed by Einarsen et al. [5], and is used internationally. The tool includes 22 items on negative behavioral experiences, 12 on bullying related to other people, 5 on bullying related to work, and 5 on bullying related to blackmail. Items are responded on a five-point Likert scale, with high scores indicating that the respondent experienced a high degree of workplace bullying. The NAQ-R is a widely used and validated tool for assessing workplace bullying. Previous studies have used the NAQ-R [5, 54] to collect quantitative data on the types of bullying experienced by nurses and their intentions to leave due to bullying. In addition, using a standardized tool such as the NAQ-R allows for comparisons of results across different studies and settings, increasing the validity and reliability of research findings.

Two other studies included in our systematic review used the NIS developed by Guidroz et al. [55]. The NIS includes 43 items measured on a five-point Likert scale. The items are grouped into five categories according to the source of incivility, as follows: general, nurses, managers, doctors, and patients/visitors. The sub-categories of general incivility include hostile climate, inappropriate jokes, and inconsiderate behavior. The sub-categories of nursing incivility include hostile climate, gossip and rumors, and free-riding. The two sub-categories of incivility by doctors and managers were abusive supervision and lack of respect [46, 55]. The purpose of the NIS tool is to provide a reliable and valid instrument for assessing incivility among hospital nurses, enabling healthcare organizations to address and mitigate this issue [55].

Two of the studies included in our systematic review used 14 and 25 items, respectively, based on Griffin’s study [31]. The variables used to measure the intervention results were bullying, experiencing symptoms of bullying, and identifying and responding to bullying. However, the effectiveness of the subcategories is limited by the number of studies with common variables. Furthermore, the NAQ-R was developed specifically to measure bullying among British workers and does not necessarily reflect the specific characteristics of other cultures [8]. Therefore, it can be inferred that there were differences between the studies regarding the effectiveness of the subcategories. This means that the effectiveness of various of the variables that featured in the reviewed studies should be measured after more relevant studies are published.

This study aimed to combine and generalize the findings of various individual studies, so a random-effects model was used. In this study, the total effect size of the cognitive rehearsal programs to address workplace bullying among hospital nurses was 0.40. This is a large and statistically significant effect size according to the standards of interpretation by Cohen [42]. The random-effect model assumes that the characteristics of the included studies, including the methods, sample, and interventions, vary and that the effect sizes are heterogeneous when estimating the effect size for the total population [35]. Thus, our study confirms the effectiveness of the analyzed cognitive rehearsal programs in addressing workplace bullying among hospital nurses. Previous studies have also validated the significance of cognitive rehearsal programs for preventing bullying in nursing contexts [32, 45, 46]. This suggests that a policy-based solution that can increase participation in such programs can be effective in solving hospital nurses’ bullying problems, and that these programs could be used to prevent and mitigate workplace bullying among hospital nurses.

Still, one of the reviewed studies reported a no significant difference between the effects of the rehearsal program and intimidation-related bullying experiences [34]. The authors of this cited study mentioned that the program encompassed 20 h of intervention. This implies that the application of a single two-hour program may have limited effects, and that the goal of the program itself may not have been superimposed into the intimidation context. This superimposition can be derived by different results obtained through periodic program application and a greater focus for the intervention on intimidation.

This study is significant because it identified a scarcity of research related to intervention programs that address workplace bullying among hospital nurses. Another reason is the lack of studies on bullying among hospital nurses. The bullying of hospital medical personnel lies in the fact that bullying cannot be considered bullying because of its vertical culture. Second, this study confirmed that cognitive rehearsal programs to address workplace bullying among hospital nurses are effective both nationally and internationally. Third, this study provides foundational data for establishing an effective intervention strategy that is better suited to the prevention of workplace bullying among hospital nurses by measuring the effects of existing programs and comparing their effect sizes.

Some limitations of this study include the small number of RCTs and study participants. Consequently, it was difficult to conduct analyses with all the subcategories included because of the lack of common variables. Moreover, only studies published in Korean and English were included; relevant studies published in other languages may have been omitted. Follow-up studies measuring the effectiveness of a variety of well-designed intervention programs on workplace bullying among hospital nurses are needed. Finally, he inclusion of multiple indicators from the same sample in a same study introduces potential overlap and weighting issues in the meta-analysis. Future research should explore methods to adjust for the influence of multiple data points from the same study to ensure a balanced analysis.

## Conclusions

Workplace bullying among hospital nurses has recently gained attention as a serious issue in South Korea. However, previous studies on workplace bullying among hospital nurses have mostly focused on outcomes, and the number of studies on interventions aimed at reducing workplace bullying among hospital nurses is insufficient. Therefore, it was not possible to determine which program was most effective. We conducted a systematic review and meta-analysis to evaluate the effectiveness of interventions for mitigating workplace bullying among hospital nurses. The findings revealed that cognitive rehearsal programs–an intervention program that incorporates aspects of cognitive behavioral therapy to address workplace bullying among nurses–are effective. Therefore, in countries where bullying occurs within nursing organizations, including the Republic of Korea, cognitive rehearsal programs should be systematically implemented to prevent bullying in hospitals.

Based on the results, the following measures were suggested: First, well-designed follow-up studies are required to measure the effects of various intervention programs on workplace bullying among hospital nurses. Second, to determine the programs that are most effective in preventing workplace bullying among hospital nurses, the effects of various intervention programs on workplace bullying among hospital nurses should be compared to understand the differences between programs.

### Supplementary Information

Supplementary Material 1.

Supplementary Material 2.

## Data Availability

All data generated or analyzed during this study are included in this published article and its supplementary information files.

## Abbreviations

CI: Confidence interval
NAQ-R: Negative Acts Questionnaire-Revised
NIS: Nurse Incivility Sale
RCT: Randomized controlled trial
RoB: Risk of bias
