# Supplementary material for: Effectiveness of cognitive rehearsal programs for the prevention of workplace bullying among hospital nurses: a systematic review and meta-analysis
Source: BMC Public Health. 2024 Jun 11;24:1568. doi: 10.1186/s12889-024-18969-x (PMC11165786; doi:10.1186/s12889-024-18969-x)
Supplement: Supplementary file 1 — Supplementary Material 1. [file 12889_2024_18969_MOESM1_ESM.docx]

**Supplementary Material 1.** Search expressions

**PubMed**

| Search | Search words/Search expressions |
| --- | --- |
| #1 | "Nurses"[MeSH] |
| #2 | "Nurses"[TW] OR "Nurse"[TW] OR "Personnel, Nursing"[TW] OR "Nursing Personnel"[TW] OR "Registered Nurses"[TW] OR "Nurse, Registered"[TW] OR "Nurses, Registered"[TW] OR "Registered Nurse"[TW] |
| #3  Combine | ("Nurses"[MeSH]) OR ("Nurses"[TW] OR "Nurse"[TW] OR "Personnel, Nursing"[TW] OR "Nursing Personnel"[TW] OR "Registered Nurses"[TW] OR "Nurse, Registered"[TW] OR "Nurses, Registered"[TW] OR "Registered Nurse"[TW]) |
| #4 | "Bullying"[Mesh] |
| #5 | "Bullying"[TW] OR "Workplace Bullying"[TW] OR "Bullying, Workplace"[TW] OR "Tae-Wom"[TW] OR "Taeoom"[TW] OR "harassment"[TW] OR "assault"[TW] OR "mobbing"[TW] |
| #6 | "Cyberbullying"[Mesh] |
| #7 | "Cyberbullying"[TW] OR "Online Bullying"[TW] OR "Bullying, Online"[TW] OR "Cyber Bullying"[TW] OR "Bullying, Cyber"[TW] OR "Virtual Bullying"[TW] OR "Bullying, Virtual"[TW] OR "On-line Bullying"[TW] OR "Bullying, On-line"[TW] OR "On line Bullying"[TW] OR "Cyber-Victimization"[TW] OR "Cyber Victimization"[TW] |
| #8 | "Workplace Violence"[Mesh] |
| #9 | "Workplace Violence"[TW] OR "Violence, Workplace"[TW] OR "Violences, Workplace"[TW] OR "Workplace Violences"[TW] |
| #10 | "Incivility"[Mesh] |
| #11 | "Incivility"[TW] OR "Rudeness"[TW] OR "Uncivil Behavior"[TW] OR "Behavior, Uncivil"[TW] OR "Behaviors, Uncivil"[TW] OR "Uncivil Behaviors"[TW] OR "Workplace Incivility"[TW] OR "Incivility, Workplace"[TW] OR "lateral violence"[TW] OR "vertical violence"[TW] OR "Horizontal Violence"[TW] |
| #12  Combine | ((((((("Bullying"[Mesh]) OR ("Bullying"[TW] OR "Workplace Bullying"[TW] OR "Bullying, Workplace"[TW] OR "Tae-Wom"[TW] OR "Taeoom"[TW] OR "harassment"[TW] OR "assault"[TW] OR "mobbing"[TW])) OR ("Cyberbullying"[Mesh])) OR ("Cyberbullying"[TW] OR "Online Bullying"[TW] OR "Bullying, Online"[TW] OR "Cyber Bullying"[TW] OR "Bullying, Cyber"[TW] OR "Virtual Bullying"[TW] OR "Bullying, Virtual"[TW] OR "On-line Bullying"[TW] OR "Bullying, On-line"[TW] OR "On line Bullying"[TW] OR "Cyber-Victimization"[TW] OR "Cyber Victimization"[TW])) OR ("Workplace Violence"[Mesh])) OR ("Workplace Violence"[TW] OR "Violence, Workplace"[TW] OR "Violences, Workplace"[TW] OR "Workplace Violences"[TW])) OR ("Incivility"[Mesh])) OR ("Incivility"[TW] OR "Rudeness"[TW] OR "Uncivil Behavior"[TW] OR "Behavior, Uncivil"[TW] OR "Behaviors, Uncivil"[TW] OR "Uncivil Behaviors"[TW] OR "Workplace Incivility"[TW] OR "Incivility, Workplace"[TW] OR "lateral violence"[TW] OR "vertical violence"[TW] OR "Horizontal Violence"[TW]) |
| #13  Combine | #3 AND #12 |
| #14  Limit | #13 AND (systematicreview[Filter]) |

**EMBASE**

| Search | Search words/Search expression |
| --- | --- |
| #1 | "nurse"/exp |
| #2 | "Nurses":ti,ab,kw,de OR "Nurse":ti,ab,kw,de OR "Personnel, Nursing":ti,ab,kw,de OR "Nursing Personnel":ti,ab,kw,de OR "Registered Nurses":ti,ab,kw,de OR "Nurse, Registered":ti,ab,kw,de OR "Nurses, Registered":ti,ab,kw,de OR "Registered Nurse":ti,ab,kw,de |
| #3  Combine | #1 OR #2 |
| #4 | "bullying"/exp |
| #5 | "Bullying":ti,ab,kw,de OR "Workplace Bullying":ti,ab,kw,de OR "Bullying, Workplace":ti,ab,kw,de OR "Tae-Wom":ti,ab,kw,de OR "Taeoom":ti,ab,kw,de OR "harassment":ti,ab,kw,de OR "assault":ti,ab,kw,de OR "mobbing":ti,ab,kw,de |
| #6 | "cyberbullying"/exp |
| #7 | "Cyberbullying":ti,ab,kw,de OR "Online Bullying":ti,ab,kw,de OR "Bullying, Online":ti,ab,kw,de OR "Cyber Bullying":ti,ab,kw,de OR "Bullying, Cyber":ti,ab,kw,de OR "Virtual Bullying":ti,ab,kw,de OR "Bullying, Virtual":ti,ab,kw,de OR "On-line Bullying":ti,ab,kw,de OR "Bullying, On-line":ti,ab,kw,de OR "On line Bullying":ti,ab,kw,de OR "Cyber-Victimization":ti,ab,kw,de OR "Cyber Victimization":ti,ab,kw,de |
| #8 | "workplace violence"/exp |
| #9 | "Workplace Violence":ti,ab,kw,de OR "Violence, Workplace":ti,ab,kw,de OR "Violences, Workplace":ti,ab,kw,de OR "Workplace Violences":ti,ab,kw,de |
| #10 | "incivility"/exp |
| #11 | "Incivility":ti,ab,kw,de OR "Rudeness":ti,ab,kw,de OR "Uncivil Behavior":ti,ab,kw,de OR "Behavior, Uncivil":ti,ab,kw,de OR "Behaviors, Uncivil":ti,ab,kw,de OR "Uncivil Behaviors":ti,ab,kw,de OR "Workplace Incivility":ti,ab,kw,de OR "Incivility, Workplace":ti,ab,kw,de OR "lateral violence":ti,ab,kw,de OR "vertical violence":ti,ab,kw,de OR "Horizontal Violence":ti,ab,kw,de |
| #12  Combine | #4 OR #5 OR #6 OR #7 OR #8 OR #9 OR #10 OR #11 |
| #13  Combine | #3 AND #12 |
| #14  Limit | #13 AND [systematic review]/lim |

**Cochrane Library**

| Search | Search words/Search expression |
| --- | --- |
| #1 | [mh "Nurses"] |
| #2 | "Nurses":ti,ab,kw OR "Nurse":ti,ab,kw OR "Personnel, Nursing":ti,ab,kw OR "Nursing Personnel":ti,ab,kw OR "Registered Nurses":ti,ab,kw OR "Nurse, Registered":ti,ab,kw OR "Nurses, Registered":ti,ab,kw OR "Registered Nurse":ti,ab,kw |
| #3 Combine | #1 OR #2 |
| #4 | [mh "Bullying"] |
| #5 | "Bullying":ti,ab,kw OR "Workplace Bullying":ti,ab,kw OR "Bullying, Workplace":ti,ab,kw OR "Tae-Wom":ti,ab,kw OR "Taeoom":ti,ab,kw OR "harassment":ti,ab,kw OR "assault":ti,ab,kw OR "mobbing":ti,ab,kw |
| #6 | [mh "Cyberbullying"] |
| #7 | "Cyberbullying":ti,ab,kw OR "Online Bullying":ti,ab,kw OR "Bullying, Online":ti,ab,kw OR "Cyber Bullying":ti,ab,kw OR "Bullying, Cyber":ti,ab,kw OR "Virtual Bullying":ti,ab,kw OR "Bullying, Virtual":ti,ab,kw OR "On-line Bullying":ti,ab,kw OR "Bullying, On-line":ti,ab,kw OR "On line Bullying":ti,ab,kw OR "Cyber-Victimization":ti,ab,kw OR "Cyber Victimization":ti,ab,kw |
| #8 | [mh "Workplace Violence"] |
| #9 | "Workplace Violence":ti,ab,kw OR "Violence, Workplace":ti,ab,kw OR "Violences, Workplace":ti,ab,kw OR "Workplace Violences":ti,ab,kw |
| #10 | [mh "Incivility"] |
| #11 | "Incivility":ti,ab,kw OR "Rudeness":ti,ab,kw OR "Uncivil Behavior":ti,ab,kw OR "Behavior, Uncivil":ti,ab,kw OR "Behaviors, Uncivil":ti,ab,kw OR "Uncivil Behaviors":ti,ab,kw OR "Workplace Incivility":ti,ab,kw OR "Incivility, Workplace":ti,ab,kw OR "lateral violence":ti,ab,kw OR "vertical violence":ti,ab,kw OR "Horizontal Violence":ti,ab,kw |
| #12 Combine | #4 OR #5 OR #6 OR #7 OR #8 OR #9 OR #10 OR #11 |
| #13 Combine | #3 AND #12 |
| #14 Limit | #13 in Cochrane Reviews |

**CINAHL**

| Search | Search words/Search expression |
| --- | --- |
| #1 | (MH "Nurses+") |
| #2 | TI ( ("Nurses" OR "Nurse" OR "Personnel, Nursing" OR "Nursing Personnel" OR "Registered Nurses" OR "Nurse, Registered" OR "Nurses, Registered" OR "Registered Nurse") ) OR AB ( ("Nurses" OR "Nurse" OR "Personnel, Nursing" OR "Nursing Personnel" OR "Registered Nurses" OR "Nurse, Registered" OR "Nurses, Registered" OR "Registered Nurse") ) |
| #3 Combine | S1 OR S2 |
| #4 | (MH "Bullying+") |
| #5 | TI ( ("Bullying" OR "Workplace Bullying" OR "Bullying, Workplace" OR "Tae-Wom" OR "Taeoom" OR "harassment" OR "assault" OR "mobbing") OR AB ( ("Bullying" OR "Workplace Bullying" OR "Bullying, Workplace" OR "Tae-Wom" OR "Taeoom" OR "harassment" OR "assault" OR "mobbing") ) |
| #6 | (MH "Cyberbullying") |
| #7 | TI ( ("Cyberbullying" OR "Online Bullying" OR "Bullying, Online" OR "Cyber Bullying" OR "Bullying, Cyber" OR "Virtual Bullying" OR "Bullying, Virtual" OR "On-line Bullying" OR "Bullying, On-line" OR "On line Bullying" OR "Cyber-Victimization" OR "Cyber Victimization") OR AB ( ("Cyberbullying" OR "Online Bullying" OR "Bullying, Online" OR "Cyber Bullying" OR "Bullying, Cyber" OR "Virtual Bullying" OR "Bullying, Virtual" OR "On-line Bullying" OR "Bullying, On-line" OR "On line Bullying" OR "Cyber-Victimization" OR "Cyber Victimization") ) |
| #8 | (MH "Workplace Violence") |
| #9 | TI ( ("Workplace Violence" OR "Violence, Workplace" OR "Violences, Workplace" OR "Workplace Violences") OR AB ( ("Workplace Violence" OR "Violence, Workplace" OR "Violences, Workplace" OR "Workplace Violences") ) |
| #10 | (MH "Incivility") |
| #11 | TI ( ("Incivility" OR "Rudeness" OR "Uncivil Behavior" OR "Behavior, Uncivil" OR "Behaviors, Uncivil" OR "Uncivil Behaviors" OR "Workplace Incivility" OR "Incivility, Workplace" OR “lateral violence" OR "vertical violence" OR "Horizontal Violence") OR AB ( ("Incivility" OR "Rudeness" OR "Uncivil Behavior" OR "Behavior, Uncivil" OR "Behaviors, Uncivil" OR "Uncivil Behaviors" OR "Workplace Incivility" OR "Incivility, Workplace" OR “lateral violence" OR "vertical violence" OR "Horizontal Violence") ) |
| #12 Combine | S4 OR S5 OR S6 OR S7 OR S8 OR S9 OR S10 OR S11 |
| #13 Combine | S3 AND S12 |
| #14 Limit | S13 Limiters - Publication Type: Systematic Review |

[**RISS**](https://www-riss-kr-ssl.proxy.cuk.ac.kr/search/detail/DetailView.do?p_mat_type=1a0202e37d52c72d&control_no=eb6ab88e55c764ccd18150b21a227875&keyword=Effects%20of%20a%20cognitive%20rehearsal%20program%20on%20interpersonal%20relationships%20%20workplace%20bullying%20%20symptom%20experience%20%20and%20turnover%20intention%20among%20nurses%20%20A%20randomized%20controlled%20trial.)

| Search | Search words/Search expression |
| --- | --- |
| #1 | "nurse"/exp |
| #2 | "burning"/exp |
| #3 | "harassment"/exp |
| #4 | "workplace violence"/exp |
| #5 | "rudeness"/exp |
| #6  Combine | #1 OR #2 OR #3 OR #4 OR #5 |

[**KISS**](https://www-riss-kr-ssl.proxy.cuk.ac.kr/search/detail/DetailView.do?p_mat_type=1a0202e37d52c72d&control_no=eb6ab88e55c764ccd18150b21a227875&keyword=Effects%20of%20a%20cognitive%20rehearsal%20program%20on%20interpersonal%20relationships%20%20workplace%20bullying%20%20symptom%20experience%20%20and%20turnover%20intention%20among%20nurses%20%20A%20randomized%20controlled%20trial.)

| Search | Search words/Search expression |
| --- | --- |
| #1 | "nurse"/exp |
| #2 | "burning"/exp |
| #3 | "harassment"/exp |
| #4 | "workplace violence"/exp |
| #5 | "rudeness"/exp |
| #6  Combine | #1 OR #2 OR #3 OR #4 OR #5 |

**KMbase**

| Search | Search words/Search expression |
| --- | --- |
| #1 | "nurse"/exp |
| #2 | "burning"/exp |
| #3 | "harassment"/exp |
| #4 | "workplace violence"/exp |
| #5 | "rudeness"/exp |
| #6  Combine | #1 OR #2 OR #3 OR #4 OR #5 |
