# Supplementary material for: Effectiveness of cognitive rehearsal programs for the prevention of workplace bullying among hospital nurses: a systematic review and meta-analysis
Source: BMC Public Health. 2024 Jun 11;24:1568. doi: 10.1186/s12889-024-18969-x (PMC11165786; doi:10.1186/s12889-024-18969-x)
Supplement: Supplementary file 2 — Supplementary Material 2. [file 12889_2024_18969_MOESM2_ESM.docx]

**Supplementary Material 2.** List of studies included in the systematic review/meta-analysis and not cited in the manuscript

Razzi CC, Bianchi AL. Incivility in nursing: Implementing a quality improvement program using cognitive rehearsal training. Nurs Forum. 2019; doi:10.1111/nuf.12366.

Stagg SJ, Sheridan D, Jones RA, Speroni KG. Evaluation of a workplace bullying cognitive rehearsal program in a hospital setting. J Contin Educ Nurs. 2011;42(9):395-403. doi:10.3928/00220124-20110823-45.
